# Novel Approaches Utilizing Metal-Organic Framework Composites for the Extraction of Organic Compounds and Metal Traces from Fish and Seafood

**DOI:** 10.3390/molecules25030513

**Published:** 2020-01-24

**Authors:** Sofia C. Vardali, Natalia Manousi, Mariusz Barczak, Dimitrios A. Giannakoudakis

**Affiliations:** 1 Institute of Biological Marine Resources, Hellenic Center of Marine Research, Agios Kosmas, Hellenikon, 16777 Athens, Greece; 2 Laboratory of Analytical Chemistry, Department of Chemistry, Aristotle University of Thessaloniki, 54124 Thessaloniki, Greece; 3 Department of Theoretical Chemistry, Institute of Chemical Sciences, Faculty of Chemistry, Maria Curie-Sklodowska University in Lublin, 20-031 Lublin, Poland; mbarczak@umcs.pl; 4 Institute of Physical Chemistry, Polish Academy of Sciences, Kasprzaka 44/52, 01-224 Warsaw, Poland

**Keywords:** metal-organic frameworks, MOFs, fish, extraction, antibiotics, antimicrobial agents, metal ions

## Abstract

The determination of organic and inorganic pollutants in fish samples is a complex and demanding process, due to their high protein and fat content. Various novel sorbents including graphene, graphene oxide, molecular imprinted polymers, carbon nanotubes and metal-organic frameworks (MOFs) have been reported for the extraction and preconcentration of a wide range of contaminants from fish tissue. MOFs are crystalline porous materials that are composed of metal ions or clusters coordinated with organic linkers. Those materials exhibit extraordinary properties including high surface area, tunable pore size as well as good thermal and chemical stability. Therefore, metal-organic frameworks have been recently used in many fields of analytical chemistry including sample pretreatment, fabrication of stationary phases and chiral separations. Various MOFs, and especially their composites or hybrids, have been successfully utilized for the sample preparation of fish samples for the determination of organic (i.e., antibiotics, antimicrobial compounds, polycyclic aromatic hydrocarbons, etc.) and inorganic pollutants (i.e., mercury, palladium, cadmium, lead, etc.) as such or after functionalization with organic compounds.

## 1. Introduction

The determination of organic and inorganic pollutants in fish tissue samples is a demanding procedure due to the complexity of the sample matrix [1]. Fish samples exhibit high protein and high fat content and therefore, their analysis is a challenging step for analytical chemists. The main problem occurring from the complexity of the sample matrix is the potential low recovery of organic and inorganic compounds that can be attributed to interactions of the analyte with endogenous food components and/or interferences from other chemical substances of the sample [2]. In order to overcome this problem, an efficient experimental protocol must be implemented for the extraction of the target analytes prior to their determination with an instrumental technique [3].

Antibiotics (including fluoroquinolones, penicillins, amphenicols, tetracyclines, sulfonamides etc.) have been widely used in farming industries to prevent bacterial infections. These chemical compounds exhibit activity against both Gram-positive and Gram-negative bacteria. Today, antibiotics are widely used for prevention and treatment of fish diseases [1,3,4,5,6,7,8,9]. The extensive use of antibiotics is a significant risk for human health which it is associated with the consumption of antibiotic residues that can directly cause allergic hypersensitivity reactions or toxic effects in humans. Moreover, their extensive use can cause an increase in antibiotic resistance in fish pathogens and potential transfer of these resistance determinants to human pathogens [3,4].

Other emerging organic contaminants that can be detected in edible fish samples include polychlorinated biphenyls and polybrominated diphenyl ethers [10], malachite green [11], polycyclic aromatic hydrocarbons [12] and food colorants [2]. The most common sample preparation techniques that are until today widely used for the extraction of organic pollutants from fish tissue are solid-phase extraction (SPE) [3,4] and liquid-liquid extraction (LLE) [13,14]. However, those conventional techniques tend to have many fundamental drawbacks since they include complicated and time-consuming steps. Moreover, they exhibit many difficulties in automation and require relatively large amounts of sample and organic solvents including ethyl acetate, chloroform, *n*-hexane, dichloromethane, etc. [15].

As an alternative to classical sample preparation approaches, various microextraction techniques including solid-phase microextraction (SPME) or liquid-phase microextraction (LPME) have been proposed. Among the most important benefits of those techniques are the consumption of less organic solvents and sample as well as the reduction of the sample treatments steps [15,16,17]. Furthermore, a wide variety of novel materials have been employed to prepare efficient sorbents for the extraction of various analytes from fish samples. These include ionic liquids (ILs) and polymeric ILs [18], graphene [19], graphene oxide [20], carbon nanotubes [21], molecularly imprinted polymers [22] and metal-organic frameworks [23].

Metal-organic frameworks (MOFs) are crystalline porous materials that consist of metal ions or clusters coordinated with organic linkers [23]. These materials are the new development on the interface between materials science and molecular coordination chemistry [24]. MOFs exhibit extraordinary properties including high porosity, tunable pore size, adjustable internal surface, high thermal and chemical stability [23,24,25]. Until today metal-organic frameworks have gained attention in a plethora of applications such as gas storage and separation [26], desulfurization of fuels [27], sensors [28], detoxification [29,30,31], catalysis [32], drug delivery and molecular imaging [33].

There are various approaches for the synthesis of metal-organic frameworks. Among them, the solvothermal approach is the most frequently used technique due to its simplicity. With this approach, the metal salt, the organic ligand and a proper solvent system are placed into a Teflon-lined vessel which is subjected to high temperature or/and pressure for a certain time span [34,35]. Other synthetic approaches involve alternative power sources like the microwave, electrochemical, mechanochemical (ball milling or ultrasonication), etc. The last year although, the synthesis of MOFs was reported either under milder conditions, for instance at less than 80 °C and near atmospheric pressure [29].

The experimental parameters during the synthesis of MOFs play a significant role to the structure and properties of the obtained material, since the level of the defect sites can be tuned [29,30]. Therefore, by controlling the quantity and the ratio of the selected metals and/or linkers, the nature and the amount of the selected solvent or the reaction temperature, pressure, and duration, it is possible to obtain MOF materials with different properties [36]. Moreover, since there is a huge variety of metal ions and organic linkers that can be used as precursors for the fabrication of MOFs, there is a nearly infinite number of MOFs that can be prepared.

In the field of analytical chemistry, MOFs have been successfully employed as adsorbents for the extraction and preconcentration of organic compounds from a wide range of samples. In the literature there are applications of MOFs for SPE [37], SPME [38], magnetic solid-phase extraction (MSPE) [39], dispersive solid-phase extraction (d-SPE) [40] etc. MOFs have been also utilized as stationary phase for gas chromatography (GC) [41], high performance liquid chromatography (HPLC) [42] and capillary electrophoresis [43]. Chiral separations with chiral MOFs as stationary phases have been also reported [44]. MOFs have also been used for the fabrication of electrochemical and fluorescent sensors for the determination of organic compounds such as antibiotics and antimicrobial agents [45,46,47,48,49,50].

MOFs have been also utilized for the extraction and preconcentration of metal ions from fish tissue. Inorganic contaminants including mercury, cadmium, lead, chromium and arsenic are dangerous contaminants in the environment, threatening human health and natural ecosystems. Water pollution led to contaminate fish with toxic metals from various sources such as use of fertilizers, discharge of industrial effluents, chemical waste agricultural drainage and domestic wastewater [51,52]. Since, those metals exhibit toxic activity even in low concentrations, it is essential to develop efficient sample preparation techniques to successfully extract and preconcentrate them from fish samples prior to their determination spectroscopically or with spectroscopic technique, including flame atomic absorption spectroscopy (FAAS) [53], electrothermal atomic absorption spectroscopy (ETAAS) [54], cold vapor atomic absorption spectroscopy (CVAAS) [55], inductively coupled plasma optical emission spectrometry (ICP-OES) [56] and inductively coupled plasma mass spectrometry (ICP-MS) [57].

In order to design a MOF material, a proper choice of its constituents should take place. In general, low-toxicity metal ions such as Fe, Mn, and Zr are preferred. Regarding the selection of metal salts, nitrates and perchlorates can be oxidized and are considered potentially explosives, while metal chlorides are usually corrosive compounds. Therefore, metal oxides and hydroxides are preferred, since they are safer and produce less hazardous by-products. Recently, the application of zero-valence metal precursors has also been introduced. As for the organic ligands, low cost carboxylic acid, such as terephthalic acid are widely used. The synthetic procedure should comply with the green chemistry principles (i.e., less steps, lower consumption of organic solvents etc). Chemometric tools are recommended for the optimization of the synthesis procedure. Finally, introduction of suitable functional groups in order to enhance the extraction efficiency and selectivity is also crucial [58].

For the real-world commercialization of MOFs and especially as a tool for analytic processes/methods, various drawbacks exist, with the most crucial to be assumed the poor chemical stability in aquatic environments, the low stability after exposure to acidic or basic solvents/solutions, the limited thermal stability as well as the difficulties in regeneration/reusability/recyclability. To strengthen MOFs’ structure and to tune them for specialized practical applications, different strategies for the design and synthesis of MOFs were showed to have a great potential, like changing/modifying the metal ions (change the oxidation state, metal ion doping) or the ligands, with the later to be the most well-explored field. For instance, ligands can be chemically functionalized either by the attachment or the insertion of specific functional groups or can be exchanged with others of different physicochemical properties and size (dimensionality) [59,60,61]. Regulating the surface properties or the structural architecture, for instance by interpenetration or formation of multi-walled frameworks and by developing inter-connection between the metal ions/clusters, are also innovative and prosperous strategies, elevating the desirable features and increasing the structural resistance against water [62]. However, these strategies can also lead to an extended presence of defect sites, as well as to the alteration of the size and the chemical environment of the pores, facts that are hard to be controlled and analysed in detail. Additionally, this kind of chemical and structural tuning upraise the complexity and the cost towards large-scale synthesis and as a result the potential of a pragmatic commercial use.

Briefly, the main approaches are the formation of composites by coating the MOFs’ particles with nanoparticles (NPs) or the growth of the framework together with the NPs. The later aspect involves the core-shell growth of the MOF around the NPs, the encapsulation of the NPs inside the final MOF nanoparticles, the individually nucleation of the MOF phase/particles in between the NPs, and the formation of macrocrystals with the NPs inside the structure as well as on the surface. In order to promote the feasibility of utilization, the establishment of easy ways to separate/obtain the MOF phase after the use are of a great demand. Towards this direction, the development of magnetic composites is a well explored and functional tactic. To achieve so, the usage of magnetic Fe-NPs has been explored in various cases. The introduction of carboxylic surface functional groups, except helping on NPs stability, is crucial for the growth of the framework. Recently, Giannakoudakis and Bandosz showed that the geometry of the framework’s structure plays a crucial role on how the growth of the MOF will occur around the nanoparticles, resulting to different effects, like the creation of mesoporosity [30,63]. Alternative approaches that will be also discussed is the coating of MOF with a polymeric layer or with bio-molecules like aptamer. Moreover, covering fibers with a layer of the active phase showed as an alternative functional approach, leading to high dispersion and availability of the active sites. Finally, the formation of graphitic/carbon-based material with incorporated NPs, derived after carbonization of functionalized MOFs, is a prosperous technique which can additionally serve as a potential way for the use of the spent samples for alternative applications [64,65,66].

A plethora of articles regarding the application of MOFs for the sample pretreatment of food, agricultural, biological, and environmental matrices can be found in the literature. Alternative approaches for increasing the MOF stability and for specifying their practical use for analytical applications is the formation of composite or hybrid materials. By this mean, simple and already widely studied MOFs as well as their composites/hybrids can be post-synthetic modified, functionalized, or immobilized on substances.

**Figure 1:**
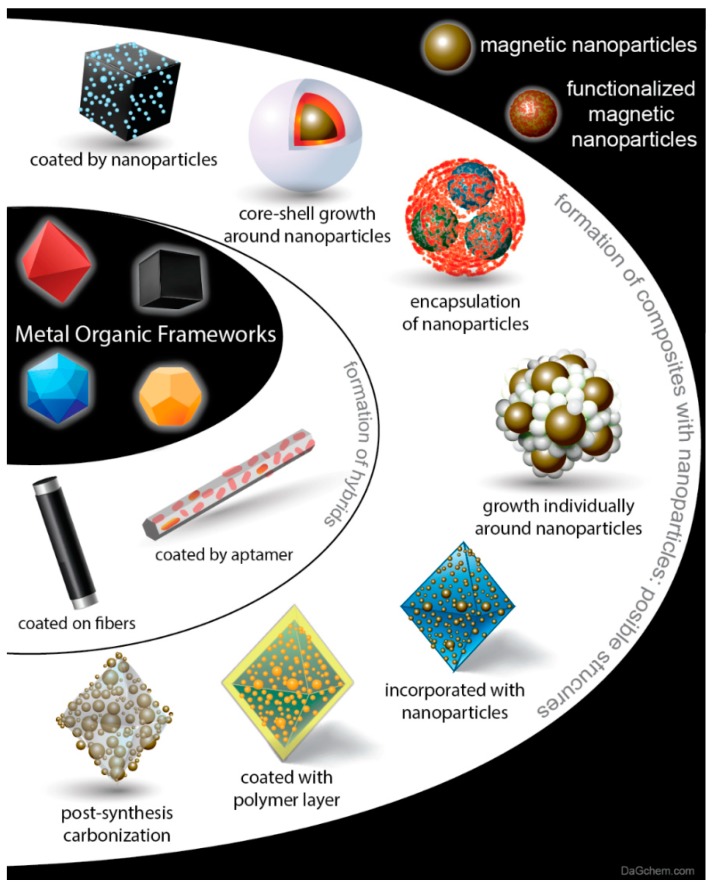
Different approaches for the formation of composite/hybrids as potential medias for the extraction, detection, or sensing of organic and inorganic pollutants from fish samples.

Herein, we aim to discuss the applications of MOFs and essentially their composites/hybrids as potential medias for the extraction, detection, or sensing of organic and inorganic pollutants from fish samples, prior to their determination with an instrumental technique. Emphasis will be given on the extraction of antibiotics as well as metals from fish tissue, since they are considered as significant contaminants of the marine environment [23,58,67,68,69,70]. All the studied in the literature cases in which MOFs were tested as extraction, detection, or biosensors media are collected in Figure 1.

## 2. Extraction and Detection of Antibiotics and Antimicrobial Agents from Fish and Seafood

### 2.1. Extraction of Antibiotics with MOFs from Seafood and Fish Samples

Fish and seafood are valued as sustenance of high nutritional value for human consumption because of their valuable fatty acid and amino acid composition. The presence of a wide range of antibiotics and antimicrobial agents has been examined in fish and seafood and a variety of analytical methods and protocols have been developed for their determination in order to satisfy the maximum residue limits (MRLs) established for the safety of consumers. Several research papers have been reported the use of the various MOF based materials (composites or hybrids) not only for the extraction of antibiotics and antimicrobials from fish and seafood and but also for their utility as fluorescent and electrochemical sensors for the sensitive detection of these compounds in fish and seafood. Table 1 summarizes the applications of novel MOF or/and their composites/hybrids for the determination of antibiotics and antimicrobials agents in seafood and fish samples, as well as for different samples for the sake of a comparison.

Mondal et al. [71] synthesized a novel polyacrylonitrile fiber coated with amino group modified MOF material, MIL-101(Cr)-NH_2_, which was used as a solid-phase micro extraction (SPME) fiber for the simultaneous determination of six antibiotics (flumequine, nalidixic acid, sulfadimethoxine, sulfaphenazole, tilmicosin and trimethoprim), representatives of four different antibiotic classes (quinolones, sulfonamides, macrolides, pyrimethamines), in the muscle tissue of living tilapia. Detection was performed using of a high-performance liquid chromatography system coupled with a tandem mass spectrometry detector (LC-MS/MS). MOF was synthesized hydrothermally by mixing chromic nitrate hydrate and 2-aminoterephthalic acid in water followed by thermal treatment (130 °C, 24 h) in an autoclave. For the preparation of the novel SPME fiber, small sized particles of MIL-101(Cr)-NH_2_ were used to form a slurry for the coating onto the surface by dip and dry of biocompatible polyacrylonitrile quartz fiber. The fiber that gave the optimum results included 50 mg of the new material. The in vivo SPME method was carried out in anesthetized fish. A hypodermic syringe was used to pierce the dorsal epaxial muscle and then the fiber was inserted in the hole for 10 min. The novel fibers were found to be stable for six sampling-desorption cycles. As for the sensitivity of the method the Limit of Detection (LOD) ranged between 0.2 ng g^−1^ to 1.1 ng g^−1^ and limit of quantification (LOQ) ranged between 0.6 ng g^−1^ and 3.7 ng g^−1^ for the six examined antibiotics. The comparison with commercial utilized fibers like C18, PDMS, PDMS/DVB composite or acrylate fiber revealed that the MOF coated fiber shower higher performances. It should be pointed out that the non-amino functionalized MOF showed dramatically lower detection capability. The novel fibers were found to be of low cost, easy to prepare, reproducible in antibiotic determination in fish muscle samples so it was assumed to be an ideal fiber for in vivo experiments [71]. As the authors concluded, the high surface area and mesoporosity, as well as the presence of the amino groups revealed to play the detrimental role.

**Figure 2:**
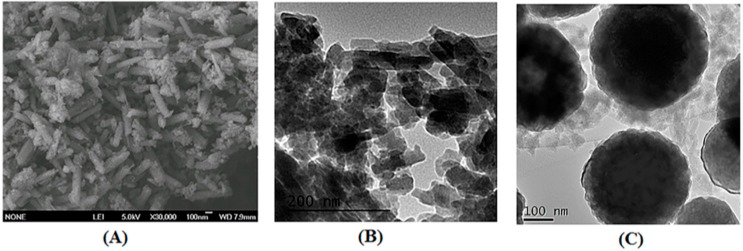
SEM and TEM images of pure JUC-48 (**A**,**B**), and TEM image of Fe_3_O_4_@JUC-48 (**C**). Adapted with permission from Reference [72]. Copyright (2017) Elsevier.

Xia et al. composed a magnetic and mesoporous MOF-based composite material as a magnetic matrix solid phase sorbent and they used it for the extraction of sulfonamides from shrimps [72]. The determination was performed by high-performance liquid chromatography (HPLC) coupled with a photodiode array detector (DAD). The Fe_3_O_4_@JUC-48 nanocomposite material was synthesized by mixing cadmium nitrate tetrahydrate, and 1,4-biphenyldicarboxylic acid with mercaptoacetic acid functionalized Fe_3_O_4_ nanoparticles. The iron oxide nanoparticles were coated with the formed rod-shaped JUC-48 crystals, with the carboxylate groups upon the modification of the NPs to act as seeds for the growth of the framework, leading to a micro-porous composite material (Figure 2).

The developed method was successfully applied except to shrimps, to a variety of samples such as chicken or pork. The LODs for all matrices were ranged from 1.73 ng g^−1^ to 5.23 ng g^−1^, while the respective LOQ values ranged between 3.97 and 15.89 ng g^−1^. Recovery rates for shrimp, pork, and chicken samples were between 76.1% and 102.6%. The novel sorbent was found to be reusable for at least seven times [72].

**Figure 3:**
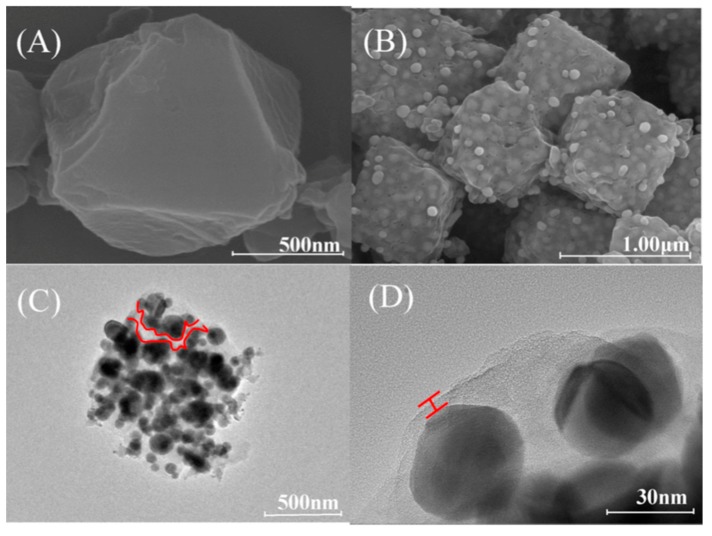
SEM images of the Cu_3_(BTC)_2_ MOF precursor (**A**), and SEM (**B**) and TEM (**C**,**D**) images of Cu@graphitic carbon composite. Adapted with permission from [73]. Copyright (2018) Elsevier.

Wang et al., reported the use of a MOF as a precursor for the synthesis of a three-dimensional (3D) porous Cu@graphitic octahedron carbon cages [73]. After the rapid room-temperature synthesis of a Cu-based metal–organic framework from copper(II) nitrate trihydrate and 1,3,5-benzene-tricarboxylic acid with the presence of ZnO nanoparticles as nucleation center (Figure 3A), the obtained material was further pyrolyzed at 700 °C under nitrogen. The final obtained material consisted of Cu nanoparticles of size 20-30 nm, encapsulated in a graphitic-carbon phase. The shape of the particles was octahedral (Figure 3B), as the one of the precursor MOF, with an open-pore structure (Figure 3C). The material showed a relatively high surface area of around 224 m^2^ g^−1^.

This composite material was used for the dispersive solid phase extraction (DSPE) of four fluoroquinolones (FQs) from fish tissue prior to their determination with HPLC coupled with a UV detector. For the extraction of FQs, a portion of 1 g of homogenized fish muscle tissues was used and after the addition of methanol the mixture was sonicated for 10 min. This method was also applied for the detection of the FQs in chicken muscle tissue as well as for water samples. The recoveries of the method in all cases were very satisfactory, ranging between 81.3% and 104.3% while the LODs were found between 0.18 ng g^−1^ and 0.58 ng g^−1^ [73].

### 2.2. Detection of Antibiotics in Seafood and Fish Samples

Except for the use of MOFs as extraction sorbents enhancing the extraction step of antibiotics, these novel materials have been used in recent years as a novel kind of fluorescent sensing materials.

Liu et al. constructed a MOF-based sensing system which had specific response to tetracyclines TC (tetracycline, chlortetracycline and oxytetracycline) antibiotics [45]. The novel luminescent MOF material (In-sbdc) was synthesized by mixing indium (III) chloride (InCl_3_) and 4,4′-stilbene-dicarboxylic acid (H_2_sbdc) in DMF-H_2_O at room temperature. The excitation and emission wavelengths that were chosen were 327 nm and 377 nm, respectively. In-sbdc showed great selectivity/specificity over other classes of antibiotics such as macrolides, chloramphenicols, aminoglycosides, β-lactams, glycopeptides, nitroimidazoles and nitrofurans. The method was applied after an easy pretreatment procedure of fish, milk, pork, or aqueous samples. The extraction of tetracyclines from fish muscle was conducted with acetonitrile by a simple solid liquid extraction (SLE) procedure. In fish, pork and milk samples recoveries ranged between 96.35% and 102.57%, while the LODs were found between 0.28 nM–0.30 nM [45].

**Figure 4:**
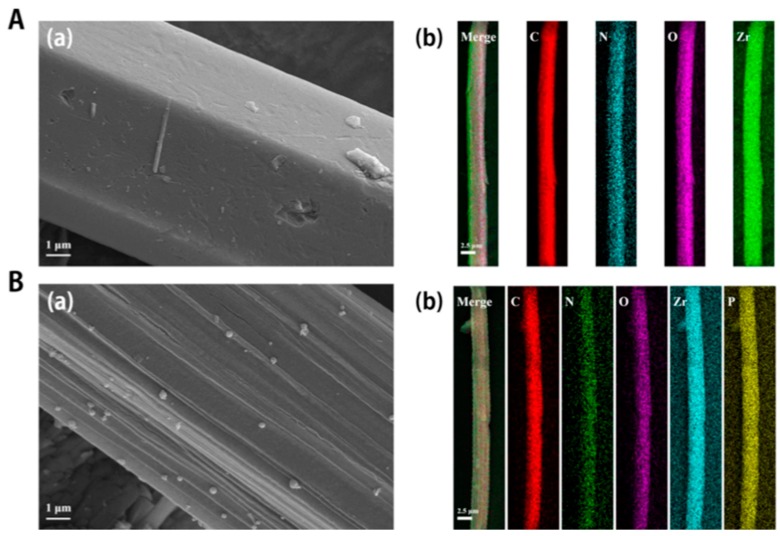
SEM images (**A**(**a**) and **B**(**a**)) and EDS mapping analysis (**A**(**b**) and **B**(**b**))) of PCN-222 (**A**(**a**) and **A**(**b**)) and of PCN-222 adsorbed with FAM-aptamer (**B**(**a**) and **B**(**b**)), with the presence of P characteristic for the homogeneous adsorption of the aptamer. Adapted with permission from [46]. Copyright (2020) Elsevier.

The same research group developed a ratiometric fluorescent sensing method for the determination of trace chloramphenicol (CAP) levels in shrimp tissues [46]. They developed a highly stable zirconium-porphyrin MOF (PCN-222) fluorescence quencher with strongly adsorbed dye-labeled Fam-aptamer due to π-π stacking, hydrogen bond and coordination interactions (Figure 4).

When CAP exists in the sample, dye-labeled aptamers are released from the surface of the novel MOF modified material, resulting in the recovery of fluorescence. The PCN-222 was prepared by dissolving zirconium (IV) chloride ZrCl_4_, tetrakis(4-carboxyphenyl)porphyrin H_2_TCPP and benzoic acid in *N*,*N*-dimethylformamide (DMF) by ultrasonication followed by the heating of mixture in an oven at 120 °C for 48 h and then at 130 °C for 24 h. The optimal conditions for the fluorescence detection of CAP was found to be the ratio of intensities I_520 nm_/I _675 nm_. The linear detection range was between 0.1 pg mL^−1^ and 10 ng mL^−1^ while the LOD was found 0.08 pg mL^−1^. The applicability of the new method for the CAP determination in shrimp samples was evaluated by a comparison with a commercial ELISA kit with the sample pretreatment proposed by the ELISA kit. The recoveries were found (91.25–104.47%) in spiked shrimp tissues and the relative standard deviation values RSD that were found 2.83%–5.02% suggested the fine accuracy and the good precision of the proposed assay [46].

Yu et al. developed an analytical method for the selective and sensitive detection of chlortetracycline (CTC) in fish muscle tissue after developing a zinc-based metal organic framework of pyromellitic acid (Zn-BTEC) [47]. The new material had the ability to enhance the aggregation-induced emission (AIE) of CTC. The new MOF material was synthesized by heating Zn-BTEC and nanometer-sized zinc oxide ZnO in DMF/H_2_O (10/1) at 180 °C for 3.5 days. The MOF after the addition CTC showed significant fluorescence enhancement at 446 nm and 540 nm which were different from the peak displacement of other tetracyclines (TCs). For the extraction of CTC by fish muscle tissue, an easy solid-liquid extraction (SLE) was used. The Zn-BTEC MOF material showed great specificity and selectivity over other antibiotics. The method was also applied in urine samples. After spiking of CTC in zebrafish samples and urine samples the recoveries ranged between 91.5% and 108.5% while the LOD of method was found 28 nM. The sensor of MOF was found to be reusable after the removal of CTC but at the cost of tedious washing [47].

The same scientific team reported the synthesis of an europium-based functional MOF with pyromellitic acid as linker, co-doped with indium (Eu-In-BTEC) [48]. The new material was applied in fluorescence sensing of doxycycline (DOX) in fish muscle tissues and urine samples. The material Eu-In-BTEC was synthesized by mixing indium nitrate hydrate, europium chloride hexahydrate and pyrometallitic acid in DMF/H_2_O (10/1 *v*/*v*) at 180 °C for 84 h. The MOF after the addition of doxycycline showed significant fluorescence enhancement at 526 nm and 617 nm. For the extraction of DOX by fish muscle tissue the same SLE pretreatment as described above was used with a homogenization of fish tissue with methanol for 5 min followed by centrifugation. The supernatants were diluted to the sensing system and mixed well before recording their emission spectra. The new MOF system could discriminate DOX from a plethora of other antibiotics such as TCs showing great specificity and selectivity. After the application of the new method in fish samples and urine samples the recoveries of DOX ranged between 105.5% and 109.5%, while the LOD was estimated 47 nM. The novel sensors indicated the specific and good performance of MOF for this kind of applications [48].

An aptamer-sensing platform was also developed for the detection of small organic molecules kanamycin and chloramphenicol using a portable fluoride-selective electrode (FSE). For this reason, the research group fabricated signal tags of nanometal-organic frameworks (NMOF) encapsulating F^−^ and labeling aptamers immobilized on one stir-bar. A double stir bar was composed to convert organic small molecules to F^−^ for signal development. The qualification of the target molecule (kanamycin or chloramfenicol) was achieved after reaction when signal tags from bar-b were washed and F^−^ was released. The preparation of signal probe (NMOF-F-@S1) was made by mixing UiO-66-COOH nanoparticles and F^−^ solution in room temperature. The double stir-bars assisted target system was prepared by the use of gold nanoparticles AuNPs. For the extraction of antibiotics from fish tissue, anhydrous sodium sulphate and ethyl acetate were added to fish muscle into a centrifuge tube. The mixture was homogenized and the supernatant was removed and transferred to a round flask. After the second extraction step with ethyl acetate the combined extract was evaporated to dryness. The residue was reconstituted by the addition acetonitrile and n-hexane and the dissolved residue was transferred into a graduated glass stopped reagent bottle and shaken. The n-hexane phase was discarded and this step was repeated with n-hexane. The acetonitrile phase was evaporated to dryness under a stream of dry nitrogen and the dry residue was dissolved in 0.5 mL of PBS (pH 7.4). The new assay was found to be very selective and sensitive in different matrices (water, milk, fish muscle, serum and urine) giving LODs between 0.35 nmol L^−1^ and 0.46 nmol L^−1^ for both antibiotics, while the recoveries ranged between 91 to 108% in all matrices for both compounds [49].

### 2.3. Extraction and Detection of Antimicrobial Agents in Seafood and Fish Samples

Malachite green (MG) and crystal violet (CV) are triphenylmethane dyes which are widely used in the aquaculture industry as antimicrobial agents due to their antifungal and antiparasitic properties in fish [77,78]. These antimicrobials have a long withdrawal period in fish and can lead to side effects, such as high toxins, high residual, carcinogenic, teratogenic, and mutation. The use of MG and CV in aquaculture remains common despite its prohibition in many countries, because of their highly effective parasiticide and fungicide [79]. A few recent applications of MOFs have been found in literature for the detection of MG and CV in fish muscle tissue.

Mohammadnejad et al. synthesized a terbium metal-organic framework (Tb-MOF), utilized as a solid phase extraction (SPE) sorbent for the extraction of MG from fish muscle tissues and water samples following by detection using a UV-Vis spectrophotometer [74]. Tb-MOF was made using the hydrothermal method by mixing Terbium(III) nitrate hexahydrate, benzene-1,3,5-tricarboxylic acid in DMF and H_2_O. Fish tissues were homogenized with the addition of hydroxylamine hydrochloride and ammonium acetate (pH 4.5 adjusted with acetic acid). A portion of the homogenate was used for MG extraction with the addition of acetonitrile followed by ultrasonication and centrifugation. The supernatant was extracted twice with acetonitrile. The solutions collected were mixed with the MOF sorbent and the mixture was stirred at room temperature for 2 h. The sorbent was separated after centrifugation and then eluted by methanol. Then the MG solution was transferred for UV-Vis analysis. After several extraction cycles Tb-MOF was found to be intact after 10 SPE cycles, showing high regeneration ability. The LOD of the method for water and fish samples was calculated 1.66 ng mL^−1^, while recoveries for fish samples and water samples ranged between 95.6% and 104.3% [74].

**Figure 5:**
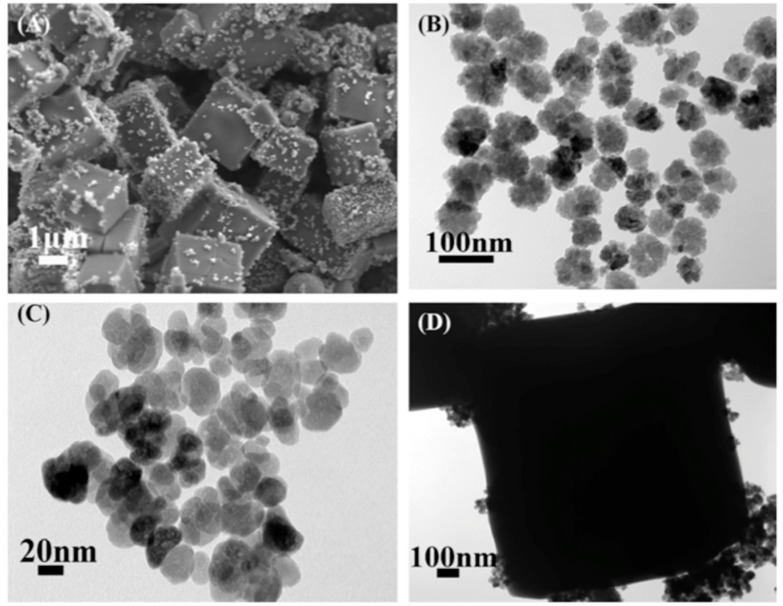
SEM image of Fe_3_O_4_@PEI-MOF-5 (**A**); TEM images of Fe_3_O_4_ (**B**), Fe_3_O_4_@PEI (**C**), Fe_3_O_4_@PEI-MOF-5 (**D**). Adapted with permission from [75]. Copyright (2018) Elsevier.

The same year, a magnetic mesoporous metal-organic framework-5 was composed by Zhou et al., for the effective enrichment of MG and CV in fish samples [75]. The developed MOF-5 material was synthesized using the solvothermal method by mixing zinc acetate hydrate, terephthalic acid and polyethyleneimine functionalized Fe_3_O_4_ nanoparticles. SEM and TEM analysis (Figure 5) revealed that the cubic particles of MOF were coated with modified Fe_3_O_4_ nanoparticles.

Fish samples were homogenized with acetonitrile and the mixture was sonicated and centrifuged. This extraction step was repeated twice. The extract was dried and dissolved in EtOH and then 10 mg of the MOF composite were added for the MSPE procedure. The mixture was stirred for 40 min and then the material was separated by a magnet and washed with 1 mL of methanol for 3 times. Malachite green and crystal violet were eluted in acidic methanol (1% formic acid) while the desorption time was 20 min. The LODs of the method for MG and CV were estimated to be 0.30 ng mL^−1^ and 0.08 ng mL^−1^, respectively while recoveries for both compounds were between 83.15% and 96.53%. The novel sorbent exhibited high magnetization, large surface area, good chemical stability and a distinctive morphology [75].

**Figure 6:**
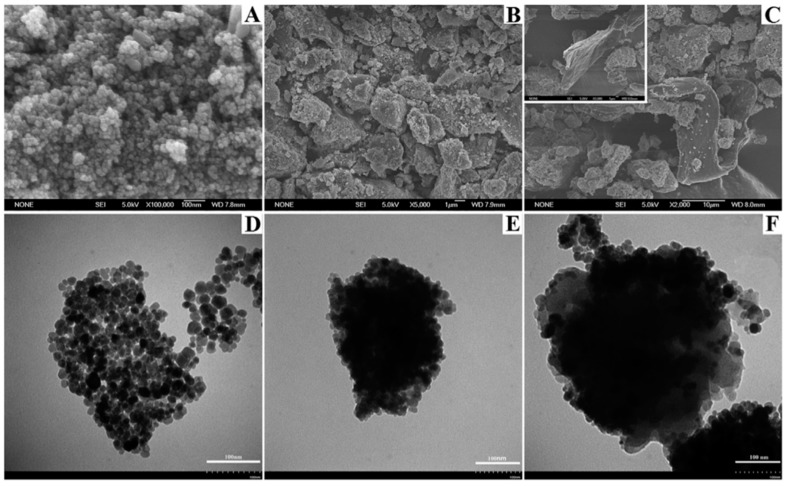
SEM images of Fe_3_O_4_ (**A**), Fe_3_O_4_@HKUST-1 (**B**), Fe_3_O_4_@HKUST-1@PDES (**C**), and TEM images of Fe_3_O_4_ (**D**), Fe_3_O_4_@HKUST-1 (**E**), Fe_3_O_4_@HKUST-1@PDES (**F**). Adapted with permission from [76]. Copyright (2019) Elsevier.

Polymeric deep eutectic solvents (PDES) functionalized amino-magnetic (Fe_3_O_4_) MOF (HKUST-1-MOF) composites (Fe_3_O_4_-NH_2_@HKUST-1@PDES) were synthesized by Wei et al. [76] and used for the selective separation of MG and CV coupled with MSPE prior to detection with UV-Vis spectrometry. For the preparation of the composite framework, spheroidal Fe_3_O_4_ nanoparticles (FeNPs) of size around 20 nm (Figure 6), were modified with 3-aminopropyltriethoxysilane (APTES). The MOF/FeNPs composite was synthesized under reflux and by mixing the amino-modified FeNPs with benzene-1,3,5- tricarboxylic acid (H_3_BTC) in a ethanol/DMF solution, followed by the addition of an aqueous copper (II) acetate monohydrate (Cu(OAc_2_)_2_·H_2_O) solution. The polymeric composite, Fe_3_O_4_-NH_2_@HKUST-1@PDES, was prepared following polymerization with deep eutectic solvents (PDES) based on 3-acrylamidopropyl trimethylammonium chloride and *N*,*N*-methylene-bisacrylamide through a seeded emulsion polymerization method.

For the sample preparation 5 g of fish samples were homogenized with 1.5 mL of 20% hydroxylamine hydrochloride and 3.5 mL of 50 mmol L^−1^ ammonium acetate for 30 min under vigorous stirring. For the MSPE procedure, the magnetic sorbent was added into the supernatant and the mixture was vortexed at room temperature to extract the dyes. The magnetic material sorbents were removed with a magnet and the supernatant was used for the detection of MG and CV by a UV-Vis spectrophotometer. Limits of detection were found to be 98.19 ng mL^−1^ for MG and 23.97 ng mL^−1^ for CV, respectively, while recoveries from fish samples for both compounds ranged between 89.43% and 100.65% [76].

A Cu-based MOF modified by silver (Ag/Cu-MOF) was fabricated by Zhou et al. [50], for the electrochemical determination of MG in fish by a Differential Pulse Voltammetry (DPV) method. The Ag/Cu-MOF material was synthesized by a one-step solvothermal synthesis from Copper(II) nitrate trihydrate, silver nitrate, and 1,3,5-benzenetricarboxylic acid through, and then it was modified on glassy carbon in order to be used as a voltammetric sensor for the detection of MG. The one-step direct synthesis was a simple and efficient. The obtained crystals showed a size distribution from tens to several hundreds of nanometers. The EDS and XPS analysis revealed an Ag to Cu elemental proportion of ~7.5%, with silver been homogeneously distributed at the entire particles.

Fish samples were homogenized after the addition of *p*-toluenesulfonic acid, hydroxylamine and acetonitrile followed by centrifugation (twice). The supernatants were collected together, dichloromethane was also added, and the solution was vortexed and centrifuged. Then, the organic phase was passed through a SCX SPE column. For the evaluation of the new developed method fish muscle samples were also analyzed with a commercial ELISA assay kit. The results showed no significant difference between the two methods suggesting the Ag/Cu-MOF modified electrode as a simple, high sensitive and accurate tool for MG determination in fish samples. Also, the detection limit of the proposed electrochemical sensor was estimated to be 2.2 nM [50].

## 3. Extraction of Metal Ions

The applications of MOFs for the extraction of metal ions from fish samples are summarized in Table 2. All fish and seafood samples were primarily digested with concentrated nitric acid for 4 h at 100 °C in Teflon beakers.

### 3.1. Extraction of Palladium

**Figure 7:**
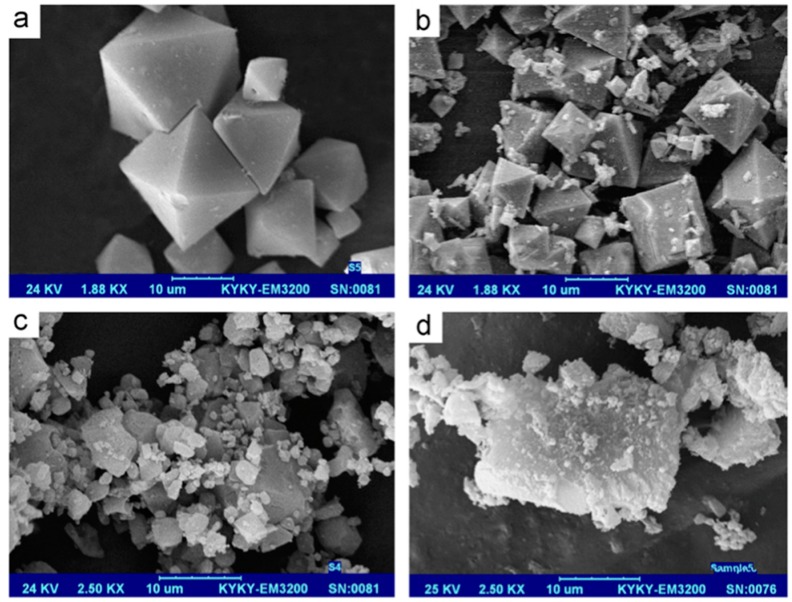
The SEM images of MOF (**a**) and magnetic MOF (**b**,**c**,**d**). Adapted with permission from [80]. Copyright (2012) Elsevier.

Palladium (Pd) is a member of platinum group metals with various scientific and technological applications. Pd has been used in metallurgy, catalysts, electronic applications, capacitors, biomedical devices, and catalytic converters for car engines [80,88,89,90,91]. This element has no biological role and its compounds are considered toxic and carcinogenic [90,91]. Due to the increasing industrial applications of palladium, it can enter the aquatic environment and it is therefore a potential danger for humans and marine life. As a result, it is important to develop efficient analytical methods for the monitoring of Pd pollution in fish samples [89,90,91]. Pd(II) has been extracted from fish samples with a magnetic metal-organic framework derived from trimesic acid and copper nitrate trihydrate [80]. The MOF was modified with pyridine functionalized Fe_3_O_4_ (Fe_3_O_4_@Py) nanoparticles in order to increase selectivity towards palladium. The crystals of original [Cu_3_(BTC)_2_(H_2_O)_3_]_n_ sample are octahedral with a smooth surface and have an average size of 10 mm (Figure 7a).

However, surface of the magnetic MOF tends to be rougher after immobilization by Fe_3_O_4_–Py (Figure 7b–d). The fish samples were initially digested with nitric acid. It was found that the optimum pH value for adsorption was 6.9. For the elution steps, the researchers used 0.01 mol L^−1^ sodium hydroxide in potassium sulfate to prevent sorbent decomposition that was observed in acidic environment. Satisfactory recovery values were observed, however no reusability data were provided. The developed method showed high sample clean-up as well as satisfactory recovery values (96.8–102.6%), however no sorbent reusability was reported.

### 3.2. Extraction of Mercury

Mercury is one of the most dangerous contaminants in the environment that threatens the human health and natural ecosystems. This element can enter the aquatic environment from a variety of sources including predominately mining and industrial production. Therefore, the accumulation of mercury in aquatic animals such as fish is unavoidable and through the dietary process, human health can be exposed to danger. Since, mercury is toxic even in low concentrations, its preconcentration from real samples is a necessary and demanding step for its determination [81,92,93].

Hg(II) has been extracted from fish samples with a porous thiol-functionalized metal-organic framework prepared from with trimesic acid and copper acetate monohydrate and functionalized with thiol-modified silica nanoparticles (SH@SiO_2_) [81]. The SH@SiO_2_ nanoparticles were prepared from silicon dioxide and (3-mercaptopropyl)-trimethoxysilane and were employed in order to increase the selectivity of the sorbent towards mercury ions. Compared to the pure MOF particles which showed an octahedral shaped particles of size 8 to 12 μm with smooth surfaces, the shape of the MOF particles in the case of the composite was no-so-well defined with rough surface covered with SiO_2_ nanoparticles. No SiO_2_ nanoparticles were detected separately and the authors reported the formation of an amorphous phase in between the MOF particles. Based on these observations and considering the XRD pattern of modified MOF (SH@SiO_2_/Cu_3_(BTC)_2_), they concluded the formation of a nanocomposite rather than a physical mixture, with the molar ratio of sulfur to copper to be 3.9 mmol g^−1^.

The prepared nanocomposite was employed for the dispersive solid-phase extraction of Hg ions from digested fish samples prior to their determination with Cold Vapor Atomic Absorption Spectrometry (CVAAS). Isolation of the sorbent was achieved with centrifugation and elution of the adsorbed analyte was performed with sodium hydroxide. It was found that this eluent provided satisfactory extraction recovery without decomposition of the sorbent. The maximum Hg(II) adsorption capacity by the nanocomposite under the optimum conditions was found 210 mg g-1, with the pseudo-second-order model to have the best fitting. However, no potential reusability of the sorbent was reported. It was indicated that although the preparation of sorbent was complicated, large quantities can be prepared at once [81].

Sohrabi prepared also an a HKUST-1 based magnetic MOF from trimesic acid and copper acetate that was modified with 4-(5)-imidazole-dithiocarboxylic acid functionalized Fe_3_O_4_ nanoparticles [82]. The modification of the synthesized MOF enhanced its selectivity towards mercury. The magnetic sorbent was used for the MSPE of mercury from fish and canned tuna samples prior to its determination with CVAAS. Compared to dispersive SPE (d-SPE), magnetic solid-phase extraction has the advantage of simple and rapid sorbent isolation with the implementation of an external magnetic field [88]. In this work, a solution of 0.01 mol L^−l^ thiourea was chosen for the elution of the adsorbed mercury ions and the sorbent was found to be reusable for up to 12 times, indicating satisfactory stability during adsorption and desorption steps.

Finally, mercury has been also extracted from fish samples with a mesoporous porphyrinic zirconium metal-organic framework (PCN-222/MOF-545) prior to CVAAS determination [83]. The sorbent was prepared from zirconyl chloride octahydrate, benzoic acid and meso-tetrakis(4-carboxyphenyl)porphyrin. Zirconium-based MOFs are known for their stability in aqueous environment. Following the strategy of use a porphyrin as linker compared to the mono-aromatic carboylic acids, the size of the pores/cages is increasing, resulting to enhanced mass transfer towards the active sites. Based on the theoretical calculations, the diameter of this MOF was estimated as 3.7 nm. The SEM micrographs revealed well-shaped hexagonal rod- and needle-like particles with diameter in the range from 300 to 800 nm, and length from 5 to 16 μm. For the pipette-tip extraction procedure, MOF was placed into a pipette-tip for the pipette-tip solid-phase extraction (PT-SPE) of Hg ions. In this work, only two milligrams of sorbent were required for the extraction of mercury. Moreover, the PCN-222/MOF-545 showed good stability under acidic conditions, since it was found to be reusable for at least 15 times after elution with HCl 10% *v*/*v*. Finally, the PT-SPE method was simple a rapid with a total extraction and desorption time span that was shorter than 7 min.

### 3.3. Multi-Element Extraction

Metal-organic frameworks have been used for the extraction of different metal ions i.e., Cd(II), Pb(II), Ni(II), Zn(II) and Co(II) from fish samples. Most of these ions are dangerous and toxic for human health. Since, those elements exist in real samples in low concentrations, their preconcentration is often considered mandatory [84,85,86,87].

Copper-(benzene-1,3,5-tricarboxylate) MOFs have been employed for the multi-element extraction of real samples after modification with Fe_3_O_4_ nanoparticles functionalized with chemical substances including pyridine (Py) [84], 4-(thiazolylazo) resorcinol (TAR) [85] and dithizone [86] in order to increase the extraction selectivity towards the target analytes. Moreover, a copper-(benzene-1,4-dicarboxylate) MOF, modified with Fe_3_O_4_ nanoparticles, functionalized with dipyridylamine has been also employed for the extraction and preconcentration of metal ions from fish samples [87]. Even though, the modified MOFs were found to provide satisfactory extraction recovery, enhancement factors and selectivity, no reusability data were reported indicating a limitation to their potential applications for the extraction of metal ions, since the Cu-based MOF are not so stable upon exposure to water. At this point we would like to mention that the utilization of the spend samples for other applications or either for the synthesis of alternative materials should gather more intense research attention, since by this strategy will close the reusability and the atom economy cycle.

Cu-BTC/HKUST-1 modified with pyridine functionalized Fe_3_O_4_ was used for the extraction of Cd(II) and Pb(II) ions from fish samples prior to FAAS determination [84]. The MOF material was stable under adsorption step (pH 6.3), however structure decomposition was noticed at the elution step with hydrochloric acid and nitric acid. Therefore, a solution of 0.01 mol L^−1^ sodium hydroxide in ethylenediaminetetraacetic acid (EDTA) was chosen as the optimum eluent. Ni(II), Cd(II), and Pb(II) ions were extracted from seafood (fish and shrimps) with a copper-(benzene-1,3,5-tricarboxylate) metal-organic framework that was modified by magnetic nanoparticles carrying covalently immobilized 4-(thiazolylazo) resorcinol (Fe_3_O_4_@TAR) [85]. Since this MOF is not stable at acidic environment, elution with EDTA was chosen. The above three ions plus Zn(II) ions were also extracted from fish samples with a copper-(benzene-1,3,5-tricarboxylate) MOF functionalized with dithizone-modified Fe_3_O_4_ nanoparticles (Fe_3_O_4_@DHz) prior to their determination with FAAS [86]. Elution of the adsorbed analytes was performed with a solution of 0.01 mol L^−1^ sodium hydroxide in thiourea to avoid any structure decomposition. Finally, in another work Cu-BTC modified with Fe_3_O_4_ dipyridylamine was employed to extract Cd(II), Pb(II), Co(II), and Ni(II) ions from fish samples prior to their determination by FAAS. Hydrochloric acid, nitric acid, sodium hydroxide, potassium sulfate, potassium chloride, thiourea and EDTA were evaluated as eluents and a solution of 0.7 mol L^−1^ EDTA in 0.13 mol L^−1^ nitric acid was chosen [87].

## 4. Extraction of Other Organic Compounds

Polycyclic aromatic hydrocarbons (PAHs) are a group of environmental contaminants consisting of two or more benzene rings fused in various arrangements. PAHs mainly come either from direct petroleum releasing or from incomplete combustion of organic materials and fossil fuel. They are persistent contaminants in environment and aquatic environment that can be transported over long distances and accumulate in living organisms, such as fish, due to their lipophilicity. By consumption of contaminated fish, the presence of PAHs is potential risk for human health, since they exhibit mutagenic, carcinogenic, teratogenic properties [94,95,96].

For the determination of PAHs in edible fish tissue samples, Hu et al. fabricated a hybrid magnetic MOF-5 with the chemical bonding approach and used it as adsorbent for the MSPE of PAHs prior to their determination with gas chromatography-mass spectrometry (GC-MS) [97]. For this purpose, MOF-5 was prepared from terephthalic acid and zinc acetate dihydrate in *N*,*N*′-dimethyl-formamide. Fish samples were initially ground and mixed with florisil and of *n*-hexane/methylene chloride (1:1, *v*/*v*). The mixture was shaken for in whirlpool bath and centrifuged, thrice. The combined extracts were dried and re-dissolved in *n*-hexane, followed by liquid-liquid extraction with sulfuric acid solution (60%, wt). After phase separation, the supernatant was collected and the magnetic sorbent was added for the MSPE procedure. Elution of the adsorbed analytes was performed with acetone. The MOF-5 sorbent was found to be stable for at least 100 extraction-desorption cycles

Polychlorinated biphenyls (PCBs) are a group of 209 chlorinated biphenyl rings with different physical-chemical properties and toxicity that depends on the number and the position of chlorine atoms [98]. PCBs are persistent organic pollutants that pose a great danger for human health and the environment because of their high toxicity and lipophilicity [99]. Fish accumulate polychlorinated biphenyls from the aquatic environment through their epithelial/dermal tissue or gills and by prey intake. PCBs can be transferred to humans via dietary intake of contaminated fish [99].

The research group of Lin and co-workers fabricated two different stir bar sorptive extraction (SBSE) bars for the extraction of polychlorinated biphenyls from fish tissue [100,101]. SBSE is an equilibrium extraction technique with large sorption phase volume that is known to provide good recovery and extraction capacity. Moreover, SBSE shows good reproducibility and low consumption of organic solvents [102]. For the first SBSE bar, a Fe_3_O_4_-MOF-5(Fe) material was used as a coating for a Nd-Fe-B permanent magnet. The bar was employed for the extraction of six PCBs from fish samples. Four different MOF materials (MIL-101(Cr), MOF-5(Zn), ZIF-8, and MOF-5(Fe) were evaluated. The results showed that Fe_3_O_4_-MOF-5(Fe) (synthesized from terephthalic acid and ferric nitrate and modified with amine-functionalized Fe3O4 nanoparticles) provided the highest extraction efficiency. Prior to the SBSE procedure, fish samples were homogenized and extracted with n-hexane. The stir bar was found to be reusable for at least 60 times with recovery values more than 80% [100]. The second SBSE bar was based on the immobilization of aptamer in the surface of MOF-5. The immobilized aptamer exhibited selectivity towards two PCBs and the stir bar was fabricated by electro-deposition. The novel sorbent exhibited high surface area as well as high selectivity [101].

Low molecular weight alkylamines (including trimethylamine and triethylamine) were extracted from salmon samples with a modified zeolitic imidazolate framework (ZIF-8) coated on SPME Arrow fiber prior to their determination with GC-MS [103]. SPME Arrow is an interesting alternative to SPME and SBSE that combines the advantages of both techniques i.e the easy automation and flexibility of SPME with the larger sorption phase volumes of SBSE. At the same time, SPME Arrow avoids the drawbacks of both techniques including the limitation in automation of SBSE and the small sorption phase volumes as well as the low fiber robustness of conventional SPME technique [103,104,105].

Lan et al., fabricated a zeolitic imidazolate framework (A-ZIF-8) and utilized as a coating material with the assistance of poly(vinylchloride) (PVC) as adhesive. Subsequently, the pore size of ZIF-8 was modified by headspace exposure to hydrochloric acid to increase the extraction efficiency for amines. Salmon samples were treated with perchloric acid prior to the extraction procedure. The developed SPME Arrow fibers exhibited good repeatability, stability and batch-to-batch reproducibility and they were found to be reusable for at least 130 times [103].

**Figure 8:**
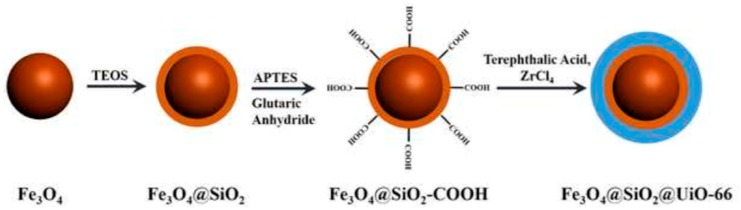
Synthetic procedure of Fe_3_O_4_@SiO_2_@UiO-66 core-shell microspheres. Adapted with permission from [106]. Copyright (2015) Elsevier.

Domoic acid, the primary amnesic shellfish poisoning toxin has been extracted from shellfish samples with a metal-organic framework magnetic nanocomposite prior to its determination by high performance liquid chromatography-tandem mass spectrometry (LC-MS/MS) [106]. For this purpose, Fe_3_O_4_@SiO_2_ microspheres were synthesized through the solvothermal approach and were treated with glutaric acid anhydride for protection and to become carboxylate terminated. Subsequently, the Fe_3_O_4_@SiO_2_ microspheres were mixed with terephthalic acid and zirconium(IV) chloride to form Fe_3_O_4_@SiO_2_@UiO-66 core-shell microspheres. The carboxylic terminal groups acted as linkers for the growth of the framework, with the entire coated iron nanoparticles acting as seeds of the MOF growth (Figure 8). Similar behavior of graphitic carbon nitride nanospheres with carboxylic terminal surface groups acting as seeds of the UiO-66 or HKUST-1 growth were reported recently by Bandosz and co-workers [29,30,60,63]. The UiO-66/g-C_3_N_4_ nanocomposite showed higher adsorption capacity of CO_2_ as well as catalytic detoxification of toxic chemical warfare agents, as for instance mustard gas. Figure 8 shows the synthetic procedure of Fe_3_O_4_@SiO_2_@UiO-66 core-shell microspheres.

**Figure 9:**
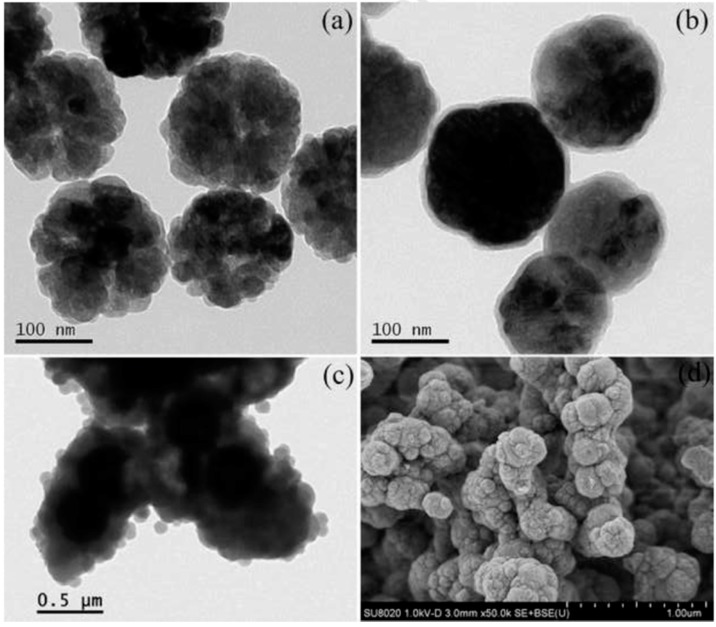
TEM images of Fe_3_O_4_ nanoparticles (**a**), after coating with SiO_2_ (Fe_3_O_4_@SiO_2_) (b), of the final nanocomposite Fe_3_O_4_@SiO_2_@UiO-66 (**c**), and SEM of Fe_3_O_4_@SiO_2_@UiO-66 (**d**). Adapted with permission from [106]. Copyright (2015) Elsevier.

The XRD pattern of the Fe_3_O_4_@SiO_2_@UiO-66 core-shell microspheres revealed to match perfectly with UiO-66 [107,108]. As revealed from the TEM micrographs (Figure 9), the Fe_3_O_4_ nanoparticles were successfully coated with a 10 nm in thickness silica layer.

For the composite material, the Fe_3_O_4_@SiO_2_ microspheres were embedded inside the final framework, with the shape of the Fe_3_O_4_@SiO_2_@UiO-66 to be spherical-shaped. The nanocomposite showed high porosity (816.3 m^2^ g^−1^ surface area and 0.533 cm^3^ g^−1^ total pore volume), with the pores to have predominately two sizes, 0.8 and 1.1 nm, characteristic as previously reported for the UiO-66. Prior to the MSPE procedure, shellfish samples were homogenized and extracted with methanol: water (1:1, *v*/*v*). Accordingly, one milligram of the magnetic sorbent was added for the extraction of domoic acid. Elution was performed with a mixture of acetonitrile:acetic acid (80:20, *v*/*v*), thrice. As a final step, the eluent was evaporated and re-dissolved in the mobile phase. Under optimum conditions, extraction recovery ranged between 93.1–107.3%, however no potential sorbent reusability was reported [106].

## 5. Extraction Mechanisms

**Figure 10:**
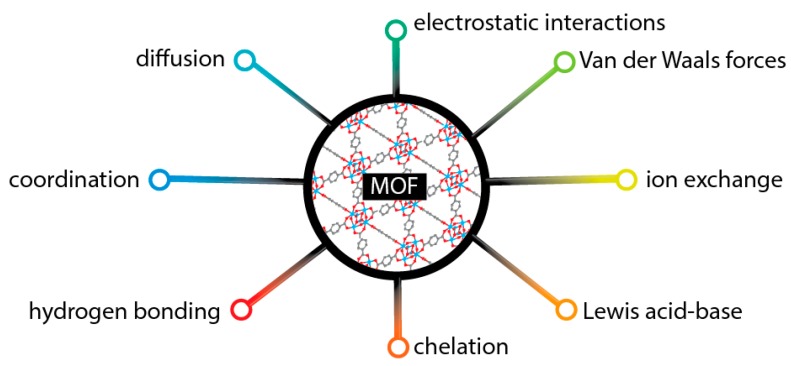
A schematic illustration of the interactions/mechanisms involved in the adsorption/extraction of organic compounds or metal ions by MOFs.

The utilization of MOFs, as well as their composites, as adsorbents for a wide range of organic compounds or metals is a well explored field [109,110,111]. Although, the herein reported and discussed articles are predominately focused on the analytic part of application, and the interpretation of the involved mechanism is limited. Figure 10 collects the most commonly reported interactions/mechanisms between MOFs and organic compounds or metal/metalloids species. It is worth to mention that more than one mechanism are involved in many cases.

For the preconcentration/adsorption of metal ions, the commonest mechanism can be assigned to Lewis acid-base interactions [112]. Pre- or post-synthetic functionalization of the framework towards the incorporation of O-, N-, or/and S-containing functional groups, is presented as a successful approach. Alternative ways of interaction occur through coordination or chelation adsorption, and functionalization, predominately of the linkers, with specific groups such as thiol, hydroxyl, or amide showed as a prosperous method for metals, while addition of -NH_2_ or -OH groups for organic compounds [113,114]. The presence of specific functional groups can positively influence also the physical-based adsorption like electrostatic interactions. The mass transfer phenomena/diffusion of the metal ions or the organic compounds thought the channels/pores towards the active sites is also an important aspect. The size and geometry of the pores is an important aspect, although the penetration of the adsorbate through the entrance/window of the porous framework is of a paramount importance [115]. A strategy to positively enhance the mass transfer is by design and synthesis of MOFs with bigger pores/cages, using larger in size linkers [116,117].

## 6. Conclusions

Metal-organic frameworks are crystalline porous materials consisting of metal ions or clusters coordinated to organic ligands forming one-, two-, or three-dimensional structures. Despite the fact that there have been only a few years since they first utilized in analytical chemistry, they have found a plethora of applications for the preparation and detection of a variety of organic and inorganic compounds in food samples. Metal-organic frameworks have been used for analytical purposes as alternative materials to conventional solid-phase extraction sorbents and they offer an interesting possibility by enriching the analytical toolbox for the easy pretreatment of fish muscle tissue and seafood samples. MOFs have also been utilized for the fabrication of electrochemical and fluorescent sensors for the selective and sensitive detection of organic compounds providing analytical techniques with very low limits of detection.

Compared with other SPE sorbents such as graphene-based nanomaterials, MOFs pose the advantage of significantly high surface area and hierarchically in structure pores/cages that result in high extraction efficiency as well as high pre-concentration factors. Therefore, MOFs have been used as adsorbents in various sample preparation techniques including SPE, SPME, d-SPE, MSPE, SBSE etc. By coupling MOFs with different micro-extraction techniques, the cost of the sample preparation and the consumption of organic solvents can be decreased and the simplicity of the pre-treatment step can be enhanced. MOFs can also be also applied for in-vivo experiments.

MOFs have been only recently utilized as sorbents for the extraction of organic compounds and metal ions from complex matrices such as fish and seafood samples. Most studies evaluate the application of functionalized MOF sorbents for the determination of specific analytes. The main advantage of metal-organic frameworks is their high total pore volume which results in high extraction recoveries. A wide variety of organic compounds including antibiotics, antimicrobial agents, polycyclic aromatic hydrocarbons, polychlorinated biphenyls etc. have been extracted with MOFs. Typically, the analytes were adsorbed from the samples and desorbed with the assistance of an organic solvents (e.g., methanol, acetonitrile etc.). Good extraction efficiency and reusability was reported in combination with low limits of detection and high enrichment factors.

Metal-organic frameworks have been also used for the extraction and preconcentration of metal ions such as palladium, mercury, cadmium, nickel, lead, cobalt etc. from fish muscle tissue and seafood samples. For inorganic analysis, adsorption was typically performed at intermediate pH value while desorption was performed with mild eluents including EDTA, sodium chloride, sodium hydroxide or thiourea. Acidic desorption was avoided since it was found to cause structure decomposition of the sorbent. However, even with mild eluents, poor reusability of MOFs for trace metals analysis has been reported. Moreover, a modification/functionalization step was required in order to enhance the selectivity of the MOFs towards the target analytes, resulting in complicated synthetic procedure for the preparation of the sorbent. Although, at the herein reported and discussed articles the explored materials as well as their performances are of a great interest, there is a luck of determine mechanistically the interactions and the features that play a key role. This is an aspect that we would like to trigger the research attention towards, since it will help to further explore different frameworks or/and to improve the performance of the already well performing ones.

In order to overcome the main drawbacks of various MOFs, like poor chemical stability in aquatic environments and in acidic/basic solutions as well as the difficulties in regeneration and reusability, various approaches have been proposed, including modifying the metal ions (i.e., by changing the oxidation state and/or by metal ion doping) or modifying the ligands (i.e., by inserting special functional groups) of the MOFs. Another strategy to overcome these drawbacks and to enhance simultaneously the adsorptive capability, is the formation of composite or hybrid novel materials. As thoroughly discussed herein, the main approaches can be summarized as coating of the MOFs’ surface with nanoparticles, core-shell growth of the framework around the nanoparticles, encapsulation of nanoparticles inside the framework, growth of the MOF phase as individual particles in between/around the utilized nanoparticles, the incorporation of the nanoparticles simultaneously inside the matrix and on the surface of the frameworks, coating of the MOF particles with different phases (like aptamer or polymeric layer), or to coat fibbers with a MOF phase.

Future perspectives can include more in-depth investigation of the use of MOFs as sorbents for the extraction of organic compounds and metal ions from fish and seafood samples, with a more intense emphasis on the determination of the involved mechanisms/interactions. Alternative or novel MOFs can be designed, synthesized, and examined for their extraction efficiency towards the desired analytes, while direct comparison for different ones will elevate the establishment of the most crucial features in each case. Furthermore, MOFs can be evaluated for their applicability after coupling with less studied sample preparation techniques such as PT-SPE and SBSE or on-line sample preparation techniques. Regarding the synthesis of MOFs, research has to be done in the field of exploring “greener” synthetic pathways, the synthesis of new generation biocompatible bio-MOFs, and application of scalable processes that could provide high quantities of MOFs, while the study of functionalization of the metal/metal clusters or of the linkers, and as a result the tuning of the pore sizes and the surface chemistry, will arise new advantageous perspectives.

## Figures and Tables

**Table 1:** Applications of MOF materials for the extraction and detection of antibiotics and antimicrobial agents in seafood and fish samples.

Matrix	Analyte	Analytical Technique	MOF Material	Sample Preparation Technique	Recovery %	LODs	Ref.
Fish (Tilapia)	Flumequine, nalidixic acid, sulfadimethoxine, sulfaphenazole, tilmicosin & trimethoprim	HPLC-MS/MS	MIL-101(Cr)NH_2_	SPME	NA	0.2–1.1 ng g^−1^	[71]
Shrimp, Chicken, pork	Sulfonamides	HPLC-DAD	Fe_3_O_4_@JUC-48	MSPE	76.1–102.6	1.73–5.23 ng g^−1^	[72]
Fish, chicken, water	Fluoroquinolones enrofloxacin, ciprofloxacin, norfloxacin, lomefloxacin	HPLC-UV	Cu based MOF	DSPE	81.3–104.3	0.18–0.58 ng g^−1^	[73]
Fish, milk, pork	Tetracyclines tetracycline, chlorotetracycline and oxytetracycline	Fluorescence Sensing	In-sbdc	SLE	96.35–102.57	0.28–0.30 nM	[45]
Shrimp	Chloramphenicol	Ratiometric Fluorescence Sensing	PCN-222	SLE	91.25–104.47	0.08 pg mL^−1^	[46]
Fish, urine samples	Chlortetracycline	Aggregation-Induced Fluorescence (AIF)	Zn-BTEC	SLE	91.5–108.5	28 nM	[47]
Fish, urine samples	Doxycycline	Fluorescence Sensing	Eu-In-BTEC	SLE	105.5–109.5	47 nM	[48]
Fish, milk, urine, serum	Kanamycin and chlortetracycline	Fluoride-Selective Electrodes (FSE)	NMOF-F^−^@Apt	SLE	91–108	0.35–0.46 nM	[49]
Fish, water samples	Malachite green	UV-Vis Spectroscopy	Tb-MOF	SPE	95.6–104.3	1.66 ng mL^−1^	[74]
Fish	Malachite green & crystal violet	UHPLC-MS/MS	Fe_3_O_4_@PEI-MOF-5	MSPE	83.15–96.53	0.30 ng mL^−1^ & 0.08 ng mL^−^	[75]
Fish	Malachite green & crystal violet	UV-Vis Spectroscopy	Fe_3_O_4_eNH_2_@HKUST- 1@PDES	MSPE	89.43–100.65	98.19 ng mL^−1^ & 23.19 ngmL^−1^	[76]
Fish	Malachite green	Differential pulse voltammetry (DPV)	Ag/Cu-MOF-modified electrode	SPE	NA	2.2 nM	[50]

**Table 2:** Application of MOFs for the extraction of metal ions from fish samples.

Analyte	Metal/Organic Linker of MOF	Modification	Amount of Sorbent (mg)/Adsorption pH/Adsorption Time(min)	Desorption Time (min)/Type of Eluent	Matrix	Sample Preparation Technique	Detection Technique	Recovery (%)	LOD (ng mL^−1^)	Reusability	Ref.
Pd(II)	Cu/Trimesic acid	Fe_3_O_4_@Py	30/6.9/6	15.5/0.01 mol L^−1^ NaOH in 9.5 (w/v %) K_2_SO_4_	*Platycephalus indicus*	MSPE	FAAS	96.8–102.6	0.37	-	[80]
Hg(II)	Cu/Trimesic acid	SH@SiO_2_	24/6/8	11/ 1.1 mol L^−1^ solution of thiourea	Fish, canned tuna	d-SPE	CV-AAS	91–105	0.02	-	[81]
	Cu/Trimesic acid	Fe_3_O_4_@4-(5)-imidazole-dithiocarboxylic acid	30/5.5/15	20/1 mol L^−1^ thiourea solution in 0.01 mol L^−1^ NaOH	*Platycephalus* *indicus*	MSPE	CVAAS	91	10	At least 12 times	[82]
	Zr/Benzoic acid and meso-tetrakis(4-Carboxyphenyl)porphyrin	-	2/5/-	-/ HCl (10% *v*/*v*)	Fish	PT-SPE	CVAAS	74.3–98.7	20 × 10^−3^	At least 15 times	[83]
Cd(II), Pb(II)	Cu/Trimesic acid	Fe_3_O_4_@Py	30/6.3/14	16.5/0.8 mol L^−l^ EDTA in 0.01 mol L^−1^ NaOH	*Platycephalus indicus*	MSPE	FAAS	92.8-97.0	0.2–1.1	-	[84]
Cd(II) Pb(II) Ni(II)	Cu/Trimesic acid	Fe_3_O_4_@TAR	50/6.2/10	15.2/0.6 mol L^−l^ EDTA	Fish, shrimps	MSPE	FAAS	83–112	0.15–0.8	-	[85]
Cd(II), Pb(II), Ni(II), Zn(II)	Cu/Trimesic Acid	Fe_3_O_4_@DHz	25/6.4/13	20/ 0.01 mol L^−1^ NaOH in thiourea	*Platycephalus indicus*	MSPE	FAAS	88–92	0.12–1.2	-	[86]
Cd(II), Pb(II), Ni(II), Co(II)	Fe/Terephthalic Acid	Fe_3_O_4_@dipyridylamine	30/6.5/11	14/ 0.7 mol L^−1^ EDTA in 0.13 mol L^−1^ HNO_3_	Fish liver, skin, muscle	MSPE	FAAS	88–108	0.13–0.75	-	[87]
